# Potential Contributions of the Tobacco Nicotine-Derived Nitrosamine Ketone to White Matter Molecular Pathology in Fetal Alcohol Spectrum Disorder

**DOI:** 10.15436/2377-1348.16.729

**Published:** 2016-07-20

**Authors:** Ming Tong, Tomas Andreani, Alexander Krotow, Fusun Gundogan, Suzanne M. de la Monte

**Affiliations:** 1 Department of Medicine, Division of Gastroenterology, and the Liver Research Center Rhode Island Hospital, Providence, RI; 2 Warren Alpert Medical School of Brown University, Providence, RI; 3 Pathobiology Graduate Program, Brown University, Providence, RI; 4 Department of Pathology, Women and Infants Hospital of Rhode Island, Providence, RI; 5 Departments of Pathology and Neurology, and the Division of Neuropathology, Rhode Island Hospital, Providence, RI

**Keywords:** Tobacco, Nitrosamines, Alcoholic brain disease, Adducts, PCR array, Myelin, Neurodegeneration

## Abstract

**Background:**

Fetal alcohol spectrum disorder (FASD) is associated with long-term deficits in cognitive and motor functions. Previous studies linked neurodevelopmental abnormalities to increased oxidative stress and white matter hypotrophy. However, similar effects occur with low-dose nitrosamine exposures, alcohol abuse correlates with cigarette smoking, and tobacco smoke contains tobacco-specific nitrosamines, including NNK.

**Hypothesis:**

Tobacco smoke exposure is a co-factor in FASD.

**Design:**

Long Evans rat pups were i.p. administered ethanol (2 g/kg) on postnatal days (P) 2, 4, 6 and/or NNK (2 mg/kg) on P3, P5, and P7 to simulate third trimester human exposures. Oligodendroglial myelin-associated, neuroglial, and relevant transcription factor mRNA transcripts were measured using targeted PCR arrays.

**Results:**

Ethanol and NNK differentially altered the expression of immature and mature oligodendroglial, neuronal and astrocytic structural and plasticity-associated, and various transcription factor genes. NNK’s effects were broader and more pronounced than ethanol’s, and additive or synergistic effects of dual exposures impacted expression of all four categories of genes investigated.

**Conclusion:**

Developmental exposures to alcohol and NNK (via tobacco smoke) contribute to sustained abnormalities in brain white matter structure and function via distinct but overlapping alterations in the expression of genes that regulate oligodendrocyte survival, maturation and function, neuroglial structural integrity, and synaptic plasticity. The results support the hypothesis that smoking may contribute to brain abnormalities associated with FASD.

## Introduction

Heavy chronic or binge alcohol exposures during development lead to sustained deficits in cognitive and motor functions. The characteristic constellation of craniofacial abnormalities together with brain structural pathology is referred to as fetal alcohol spectrum disorder (FASD)^[1,2]^. The cerebellum, temporal lobe, and white matter are major targets of ethanol’s teratogenic effects, and while there is good evidence that ethanol-induced pathology in the cerebellum and temporal lobe is linked to impaired insulin and insulin-like growth factor type 1 (IGF-1) signaling through Akt-mediated growth, cell survival, metabolic, and neuronal plasticity networks, the nature and pathogenesis of white matter hypotrophy are poorly understood. However, since growth factor, including IGF-1 signaling through phosphatidylinositol-3-kinase (PI3K) and Akt promotes oligodendrocyte survival and function, including myelin synthesis, maintenance, and homeostasis^[3–5]^, impairments in insulin/IGF signaling that occur with alcohol exposure could potentially underlie the white matter abnormalities in FASD. Since the white matter hypotrophy may be due to reduced myelin synthesis, maturation and, maintenance or primary loss of axons, evaluating the state of oligodendrocyte function could aid our understanding of its molecular pathogenesis.

Oligodendroglia produces central nervous system (CNS) myelin. Myelin has an abundant lipid composition (70 – 85%) with lower proportions of proteins (15 – 30%) and water compared with other cellular membranes. High lipid content ensures optimum nerve cell conduction. Oligodendrocytes develop from precursor cells (OPC 1–3) that differentially express Cnp, Ng, PDGF-Rα, Olig2, Dix2, and Nkx. OPCs differentiate into immature oligodendroglia that express Cnp, Olig1, and low levels of Olig2, which mature and express Cnp, Olig1, low Olig2, and RTN4^[6–8]^. Finally, mature myelinating oligodendroglia further express distinct integral membrane proteins including, myelin basic protein (MBP), myelin associated glycoprotein (MAG), myelin oligodendrocyte glycoprotein (MOG), and proteolipid protein (PLP)^[3,4,9]^. Oligodendroglial differentiation is regulated by Wnt/β-catenin (canonical pathway) via the Tcf4 transcription factor^[10]^, Notch1-HES5, and insulin/IGF signaling^[11–19]^.

Chronic ethanol exposure delays oligodendroglial maturation, expression of MBP^[20]^ and MAG^[21]^, and de novo myelin synthesis^[22]^. White matter integrity is crucial for CNS function since reduced brain myelination during development results in cognitive impairment^[23]^. Mixed glial cultures were used to demonstrate oligodendrocyte’s extreme vulnerability to the toxic effects of ethanol compared with other cell types. The pro-death effects of ethanol are mediated by inhibition of PI3K and Erk MAPK^[24]^. Ethanol impaired signaling through PI3K/Akt is due to reductions in insulin/IGF receptor binding[25] associated with altered membrane lipid composition^[25]^ and fluidity ^[26]^, as well as decreased phosphorylation and activation of downstream pathways^[27–31]^.

Despite evidence that ethanol exposures can be sufficient to cause FASD, variability in the nature and severity of the phenotype suggests that co-factors may contribute to its pathogenesis. Of note is that a very high percentage of heavy drinkers (up to 80%) also abuse tobacco products, mainly by smoking cigarettes^[32]^. Review of an outpatient substance abuse treatment center database for pregnant women revealed that from 2010 to 2013, 74% of the pregnant alcohol users (N = 57) smoked cigarettes compared with 42% of controls (N = 31) (P = 0.0053)^[33]^. Despite strong evidence linking cigarette smoking during pregnancy to impaired fetal growth and development, and neurocognitive function^[34]^, the mechanisms are not well understood. Given the frequency of overlapping exposures and their known independent adverse effects on development, further research is needed to determine the degree to which alcohol and tobacco exposures produce differential or additive adverse effects on white matter in the immature brain.

Although the vast majority of research designed to investigate alcohol-tobacco interactive effects has been focused on carcinogenesis^[35–38]^, particularly in relation to the tobacco-specific nitrosamine, 4-(methylnitrosamino)-1-(3-pyridyl)-1-butanone (NNK) and its metabolites^[36]^, it is known that low, sub-mutagenic exposures to nitrosamines such as streptozotocin, N-nitrosodimethylamine (NDEA), and NNK cause insulin resistance with impaired signaling through PI3K-Akt, increased DNA damage, lipid peroxidation, mitochondrial dysfunction, and ER stress^[39–42]^. The present study tests the hypothesis that sub-mutagenic exposures to NNK are sufficient to cause white matter molecular pathology similar to the effects of ethanol, and possibly exacerbate the adverse effects of ethanol with respect to impairments in oligodendrocyte and neuroglial gene expression.

## Methods

Long Evans rat pups were divided into 4 groups and administered 50 μl i.p. injections of: saline vehicle as control; pharmaceutical grade ethanol (2 g/kg in saline); NNK (2 mg/kg in saline); and ethanol + NNK. Ethanol treatments (binge) were administered on postnatal days (P) 2, 4, 6, and 8^[43–45]^, and NNK was administered on P3, P5, P7, and P9. These models simulated 3rd trimester-equivalent human pregnancy exposures to alcohol and tobacco toxins. The rats were sacrificed at 6 weeks of age to examine long-term effects on temporal lobe insulin/IGF-1 signaling through Akt growth and metabolic pathways during late adolescence. All experiments were performed in accordance with protocols approved by Institutional Animal Care and Use Committee at the Lifespan-Rhode Island Hospital, and they conformed to guidelines established by the National Institutes of Health.

Targeted quantitative RT-PCR arrays were used to measure expression of genes linked to oligodendrocyte and astrocyte function (**“S Table 1”**). The objective was to evaluate ethanol and NNK effects on genes relevant to CNS myelin and white matter development and integrity. PCR primer pairs were designed with MacVector 12 (Cary, NC) and Primer 3 (http://primer3.sourceforge.net/) software (**“S Table 2”**). Targeted arrays were constructed by spotting and drying primer pairs (100 nmol/5 μl) into individual wells of a Light cycler 480 Multi-well Plate 96 (Roche, Indianapolis, IN). The arrays were sealed and stored at −80°C. On the day of use, the array plates were thawed on ice and 20 μl of reaction cocktail containing cDNA from 5 μg RNA template and Sybr green master mix were added to each well. PCR reactions were performed in a Roche Lightcycler 480 System. Gene expression was analyzed using the ΔΔCt method with results normalized to housekeeping control genes (hypoxanthine-guanine phosphoribosyltransferase, HPRT; and Ribonuclear protein, RNP).

Each experimental group included 8 – 10 rats. Inter-group comparisons were made using two-way analysis of variance (ANOVA) with Tukey or linear trend post hoc tests (GraphPad Prism 6, San Diego, CA). F-ratios and P-values are tabulated. Significant post-test differences and trends (0.05 < P < 0.10) are shown in the graphs. Heat maps were constructed using Version 3.1 of R software. Exploratory analysis identified missing values verified the quality of observed data. To scale the data, row means were subtracted from each cell. The resulting values were further divided by the standard deviation in order to obtain a z-score of each individual cell. Row values are scaled to have a mean = 0 and standard deviation (S.D.) = 1. The resulting values were plotted using a cosmetically modified heatmap library function in R and a 6-color palette. We also applied hierarchical clustering algorithm using Euclidean distance function on the overall table to display a dendrogram of mRNAs.

## Results

### PCR Targeted Array Studies

PCR arrays were generated to measure expression of 84 genes involved in glial and neuronal growth, maturation, and function. Pilot screening enabled us to eliminate 55 genes due to their low levels of expression or absent inter-group differences. The follow-up studies were focused on 29 genes (Supplementary Tables 1 and 2) in which mRNA levels were measured by quantitative real time PCR using the SA Biosciences protocol (Qiagen, Carlsbad, CA). Gene expression was calculated using the ΔΔ-Ct method with results normalized to HPRT. Data were analyzed by two-way ANOVA with the post hoc Tukey multiple comparisons test. In addition, functionally clustered summary results are depicted in hierarchical heatmaps that were generated in R.

### Oligodendroglial Genes

As markers of immature oligodendroglia, we measured nestin, vimentin, PROM1, CNP, PDGFR-α, GC, and GalC, and for mature myelinating oligodendroglia, we measured PLP, MOG, MAG-1, MBP, RTN4, RPAIN, and ST8sia1 expression (Table 1). Significant ethanol effects were observed with respect to PROM1, GC, GalC, MAG-1, RPAIN, and ST8sia1, and trend effects were observed for nestin and PLP. Significant NNK effects were detected for vimentin, PROM1, PDGFR-α, GC, GalC, PLP, MOG, MBP, RTN4, RPAIN, and ST8SIa1, and trend effects occurred for CNP and MAG-1 expression. Significant ethanol x NNK interactive effects occurred for PROM1, GalC, PLP, MBP, and RPAIN, and trend effects were observed for GC.

**Figure 1:**
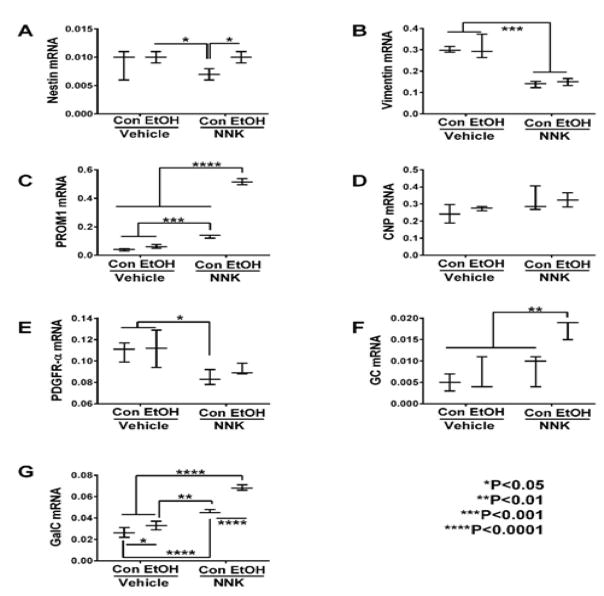
Ethanol and NNK effects on temporal lobe expression of immature oligodendroglial genes. mRNA levels of (A) Nestin, (B) Vimentin, (C) PROM1, (D) CNP, (E) PDGFR-α (F) GC, and (G) GalC, were measured using targeted PCR arrays. Gene expression was calculated using the ΔΔCt method with results normalized to HPRT1. Inter-group comparisons were made by two-way ANOVA (Table 1) with the post-hoc Tukey multiple comparison test.

**Figure 2:**
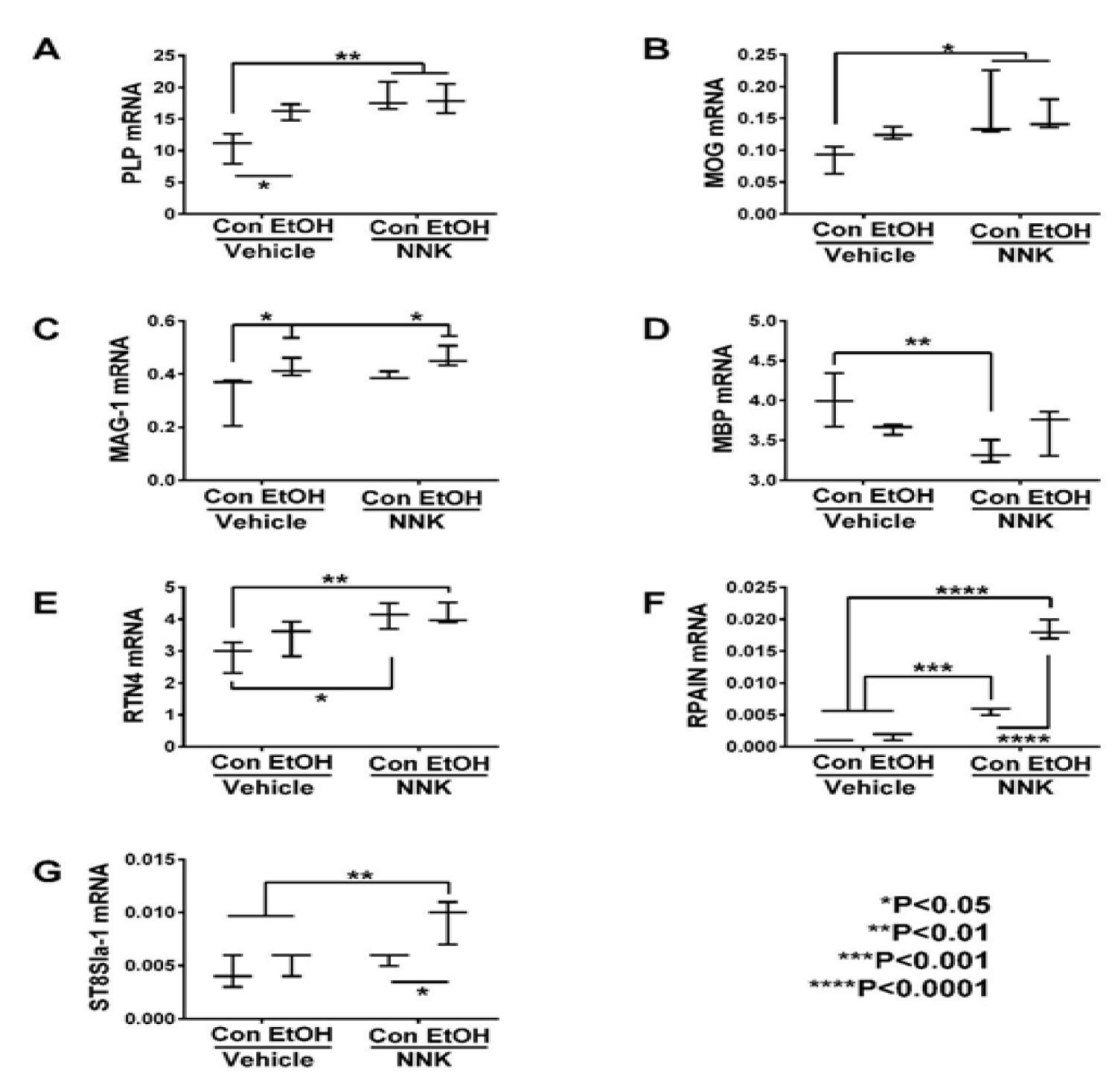
Ethanol and NNK effects on temporal lobe expression of mature oligodendroglial genes. mRNA levels of (A) PLP, (B) MOG, (C) MAG-1, (D) MBP, (E) RTN4, (F) RPAIN, and (G) ST8SIa-1 were measured using targeted PCR arrays. Gene expression was calculated using the ΔΔCt method with results normalized to HPRT1. Inter-group comparisons were made by two-way ANOVA (Table 1) with the post-hoc. Tukey multiple comparisons test.

Graphed results and post hoc repeated measures tests revealed significant intergroup differences with respect to all immature and mature myelin-associated genes except for CNP (Figure 1D). Ethanol’s significant effects were restricted to one immature (GalC) (Figure 1G) and two mature i.e. PLP (Figure 2A) and MAG-1 (Figure 2C) genes that were all increased relative to control. However, there were also statistical trend-wise increases in MOG (Figure 2B) and reductions in MBP (Figure 2D) relative to control.

NNK and particularly ethanol + NNK had broader effects on the expression of both immature and mature oligodendroglial genes, and in several instances, combined exposures produced additive or synergistic responses. NNK significantly increased GalC (Figure 1G), PROM1 (Figure 1C), PLP (Figure 2A), MOG (Figure 2B) RTN4 (Figure 2E), and RPAIN (Figure 2F), and had trend effect increases on MAG-1 (Figure 2C), but significantly decreased nestin (Figure 1A), vimentin (Figure 1B), PDGFR-α (Figure 1E) and MBP (Figure 2D) relative to control, ethanol, or ethanol + NNK. Ethanol + NNK significantly increased GC (Figure 1F), GalC (Figure 1G), PROM1 (Figure 1C), PLP (Figure 2A), MOG (Figure 2B), RTN4 (Figure 2E), RPAIN (Figure 2F) ST8Sia-1 (Figure 2G), and MAG-1 (Figure 2C), and decreased vimentin (Figure 1B) relative to control. Additive or synergistic stimulatory effects of the dual treatments were observed with respect to GC (Figure 1F), GalC (Figure 1G), PROM1 (Figure 1C), RPAIN (Figure 2F) and ST8SIa-1 (Figure 2G).

### Oligodendroglial Heatmaps

**Figure 3:**
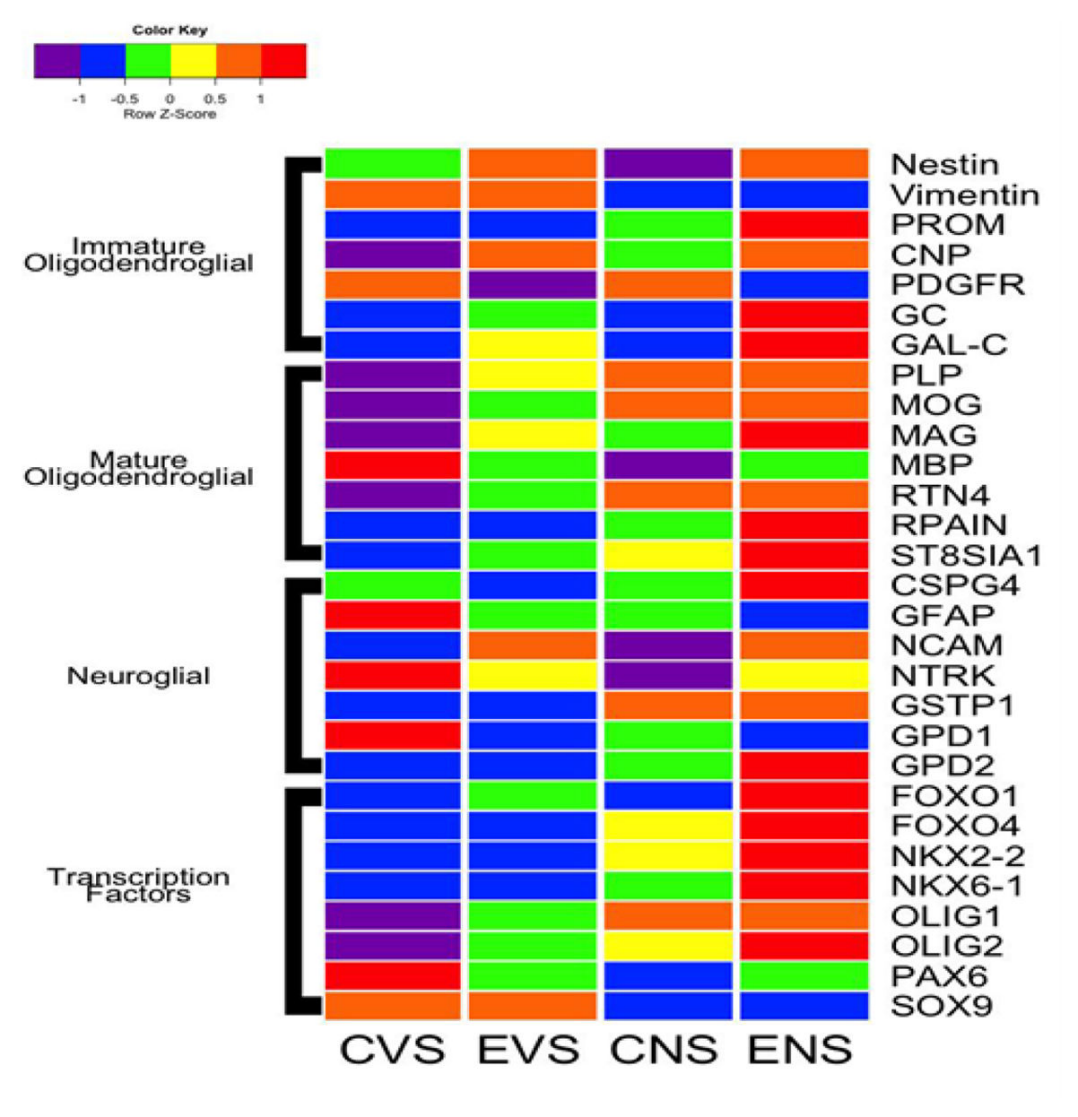
Heatmap with grouping according to gene function. The heatmap was generated using Version 3.1 of R software. Results shown with the 6-color palette correspond to z-scores, which were scaled to have a mean of 0 and S.D. of 1. Results were sorted to juxtapose data corresponding to immature oligodendroglial genes, mature oligodendroglial genes, neuroglial genes, and transcription factors. CVS = control diet with vehicle treatment and saline binge treatment; EVE = ethanol diet with vehicle treatment and ethanol binge; CNS = control diet with NNK treatment and saline binge treatment; ENE = ethanol diet with NNK treatment and ethanol binge.

Heatmaps corresponding to immature and mature oligodendroglial gene expression are depicted in Figure 3. Among the immature oligodendroglial genes, ethanol increased expression of Nestin, CNP, GC, and GalC but decreased PDGR-α. The dual ethanol + NNK treatments produced similar (Nestin, CNP, PDGFR-α), more exaggerated (GC, GAL-C) responses occurred following ethanol + NNK treatments. NNK decreased expression of Nestin and Vimentin, increased PROM1 and CNP, and had no detectable effect on PDGFR-α, GC, or GalC (Figure 3). In essence, ethanol and NNK differentially altered expression of immature oligodendroglial genes, whereas the dual exposures mimicked effects of either or were synergistic (PROM1, GC, GalC).

With regard to mature oligodendroglial genes, ethanol- and NNK-mediated patterns of altered gene expression were more consistent. MBP expression was highest in control brains, and prominently reduced by all other treatments. Otherwise, the expression levels of most genes increased with ethanol, NNK, and ethanol + NNK treatments. For PLP, MOG, RTN4, RPAIN, and ST-8SIA1, gene expression increased from ethanol to NNK. Except for MBP, mature oligodendroglial gene expression was highest in the ethanol + NNK group (MAG, RPAIN, ST8SIA1) or similarly high in the NNK and ethanol + NNK groups (PLP, MOG, RTN4).

### Neuroglial Genes

**Figure 4:**
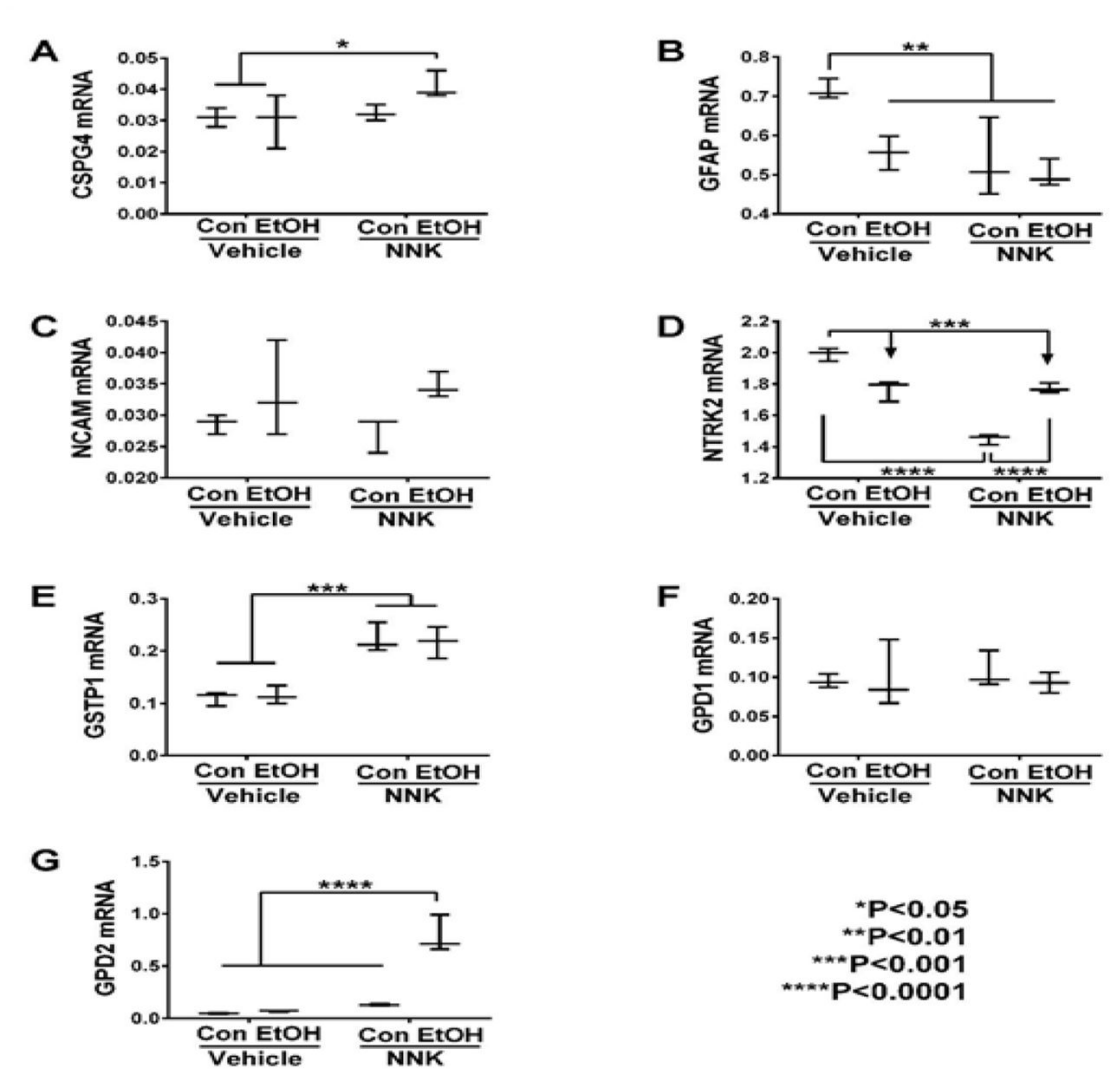
Ethanol and NNK effects on neuronal/glial gene expression in the temporal lobe. (A) CSPG4, (B) GFAP, (C) NCAM, (D) NTKR2, (E) GSTP1, (F) GPD1, and (G) GPD2, mRNA levels were measured using custom targeted PCR arrays. Intergroup comparisons were made by two-way ANOVA (Table 2). Inter-group comparisons were made by two-way ANOVA (Table 2). Tukey post tests assessed differential effects of ethanol and NNK.

The neural-glial mRNA transcripts measured included: CSPG4, GFAP, NCAM, NTRK2, GSTP1, GPD1 and GPD2. Two-way ANOVA tests demonstrated significant effects of ethanol on GFAP, NCAM, and GPD2, and trend effects on NTRK2 (Table 2). NNK had significant effects on GFAP, NTRK2, GSTP1, and GPD2, and trend effects on CSPG4. Ethanol x NNK interactive effects were significant for NTRK2 and GPD2, and had a trend effect on GFAP. Post hoc tests revealed that ethanol, GFAP (Figure 4B) and NTRK2 (Figure 4D) relative NNK, andethanol + NNK significantly to control, while NNK and ethanol + NNK increased GSTP1 relative to control and ethanol groups (Figure 4E), ethanol + NNK increased CSPG4 relative to control and ethanol (Figure 4A), and GPD2 relative to the other three groups (Figure 4G). No significant treatment effects occurred with respect to GPD1 (Figure 4F) or NCAM (Figure 4C).

### Transcription Factors

**Figure 5:**
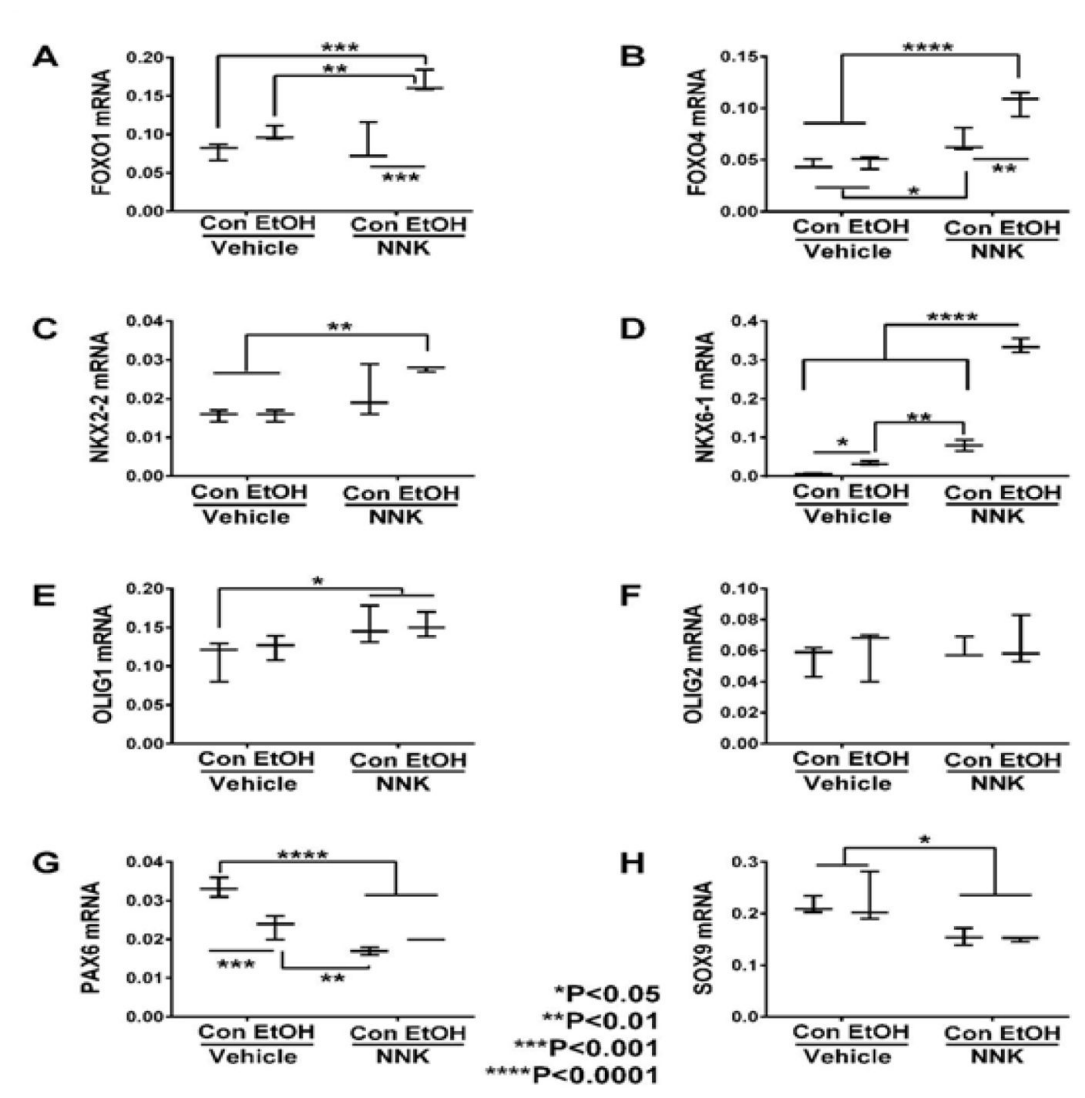
Ethanol and NNK effects on temporal lobe expression of transcription factor genes. The mRNA levels of (A) FOXO1, (B) FOXO4, (C) NKX2-2, (D) NKX6-1, (E) Olig1, (F) Olig2, (G) PAX6, and (H) SOX9 were measured using targeted PCR arrays, and results were calculated using the ΔΔCt method after normalizing to HPRT1. Intergroup comparisons were made by two-way ANOVA (Table 2) with the post-hoc Tukey multiple comparison test.

Glial-associated transcription factor mRNA transcripts measured included: FOXO1, FOXO4, NKX2-2, NKX6-1, Olig1, Olig2, PAX6, and SOX9. Two-way ANOVA tests demonstrated significant ethanol and ethanol x NNK interactive effects on FOXO1, FOXO4, NKX6-1, and PAX6, and NNK effects on all genes except Olig2 (Table 2). Post hoc tests revealed that ethanol significantly increased expression of NKX6-1 (Figure 5D) and decreased PAX6 (Figure 5G) relative to control. NNK significantly increased expression of FOXO4 (Figure 5B), NKX6-1 (Figure 5D) and Olig1 (Figure 5E), and decreased PAX6 (Figure 5G) and SOX9 (Figure 5H) relative to control and ethanol-exposed samples. Ethanol + NNK significantly increased expression FOXO1 (Figure 5A), FOXO4 (Figure 5B), NKX6-1 (Figure 5D), OLIG1 (Figure 5E), and NKX2-2 (Figure 5C), and decreased expression of PAX6 (Figure 5G) and SOX9 (Figure 5H) relative to the control, ethanol, and/or NNK groups. Stepwise increases in gene expression from control or ethanol, to NNK, then ethanol + NNK occurred for FOXO4, NKX6-2, OLIG1, and NKX2-2, and similar trend reductions occurred for PAX6 and SOX9; these responses reflect additive effects of ethanol and NNK in the dual-treatment group.

### Neuroglial Heatmap Results

Heatmap analysis demonstrated that control temporal lobes had high mRNA levels of GFAP, NTRK, and GPD1, intermediate levels of CSPG4, and low levels of NCAM, GSTP1, and GPD2 (Figure 3). Ethanol inhibited expression of CSPG4, GFAP, NTRK, and GPD1, maintained low levels of GSTP1 and GPD2, and increased expression of NCAM. NNK’s effects differed entirely from those of ethanol, except that GFAP which was expressed at similar levels. Ethanol + NNK reversed the expression profiles observed in control samples, resulting in lower levels of GFAP, NTRK, and GPD1, and higher levels of CSPG4, NCAM, GSTP1, and GPD2. NNK + ethanol’s effects mimicked responses of ethanol- (NCAM, NTRK and GPD1) or NNK treated (GSTP1) samples, or else they appeared to be synergistic (CSPG4, GFAP, GPD2).

### Transcription Factor Heatmap Results

Very low levels of FOXO1, FOXO4, NKX2- 2, NK6-1, Olig1 and Olig2, and high levels of PAX6 and SOX9 in control temporal lobes, and sharply opposite trends in the ethanol + NNK group (Figure 3). Ethanol treatments modestly increased expression of FOXO1, Olig1, and Olig2, reduced expression of PAX6, and caused no change in FOXO4, NKX2-2, NKX6-1, or SOX9 relative to control.

NNK’s effects were intermediate between those of ethanol and ethanol + NNK (FOXO4, NKX2-2, NKX6-1, Olig2), similar to those of ethanol + NNK (Olig1, SOX9), similar to control (FOXO1), or different from the other groups (PAX6).

## Discussion

Ethanol and Tobacco Nitrosamine (NNK) Related Brain Disease: This study was designed to examine the independent and interactive effects of ethanol and NNK exposures on neurodegeneration in adult rats. The chronic-binge ethanol exposure model was used to mimic the human clinical scenario that is most prevalent among alcoholics. Chronic low-dose NNK tobacco-specific nitrosamine exposure effects were of interest in relation to alcoholic neurodegeneration because: 1) a very high percentage of heavy drinkers also smoke^[32]^; 2) the causes of progressive alcoholic brain disease are more complex than alcohol consumption per se; 3) previous studies showed that exposures to sub-mutagenic levels of other nitrosamines cause neurodegeneration^[39–41,46]^; and 4) we recently showed that early developmental exposures to ethanol and dietary nitrosamines used in preserved and processed foods, i.e. N-nitrosodiethylamine, differentially or additively contributed to cerebellar abnormalities associated with fetal alcohol spectrum disorder^[47]^. Our working hypothesis herein was that chronic low dose NNK exposures mediate CNS degenerative abnormalities that overlap with and exacerbate adverse effects of alcohol. NNK rather than tobacco smoke effects were studied because smoking could confound the results by causing pulmonary disease^[48,49]^.

### Immature oligodendroglial genes

The results show that ethanol had limited effects on the expression of immature oligodendroglial genes and only increased GalC, whereas NNK, with or without ethanol co-exposures significantly modulated expression of 6 of the 7 genes examined. Stimulatory effects occurred for GC, PROM1 and GalC, while inhibitory effects occurred with respect to nestin, Vimentin, and PDGFR-α. Nestin regulates vimentin intermediate filament disassembly during growth and is needed for survival, renewal and proliferation of neural progenitor cells. Inhibition of Nestin corresponds with prior reports of impaired neuronal genesis in chronic ethanol-exposed experimental animals^[50,51]^, illustrating how NNK’s effects can mimic those of ethanol.

PDGFR-β is a cell surface receptor tyrosine kinase expressed in oligoprogenitor cells and when stimulated with PDGF, activates proliferation pathways. Therefore, NNK inhibition would have impaired growth of immature oligodendroglia. GC is a Vitamin D binding nuclear hormone receptor that interacts with RXR and regulates maturation of immature oligodendroglial precursors into myelin-generating oligodendrocytes^[52,53]^. The significantly elevated levels in ethanol + NNK exposed temporal lobes suggest that the dual treatments may lead to accelerated maturation of oligodendroglia. At the same time, the increased expression of PROM1, particularly in the ethanol + NNK group could have led to suppressed maturation of progenitor or stem cells^[54,55]^, reducing the pool of myelin-generating precursors. GalC (galactosylceramidase/galactocerebrosidase) hydrolyzes the galactose ester bond of galactosylceramide in its lysosomal catabolism, and either over-expression^[56]^ or deficiency^[57]^ can adversely affect oligodendrocyte maturation and function.

### Mature oligodendroglial markers

Gene expression and heatmap analyses of mature oligodendroglial gene expression profiles revealed progressive up-regulation of PLP1, MOG, MAG-1, RTN4, RPAIN, and ST8SIA1 from control, to ethanol, and then NNK exposures, and either further increases with dual exposures, or similar maximum levels of expression as observed in either ethanol or NNK-exposed brains. The generally higher levels of both immature and mature myelin gene expression in ethanol + NNK exposed brains relative to the other groups most likely reflects responses to injury in the early understand how these responses impact white matter structure and function in adolescence. MBP was the exception to these trends in that the highest expression levels were in control brains. Ethanol and NNK conspicuously down-regulated MBP relative to control. Since MBP is a major protein constituent of myelin sheaths and has an important role in forming and stabilizing myelin membranes, irrespective of the up-regulation of other myelin genes, MBP’s down regulation would impair the integrity of mature white matter myelin.

### Neuroglial genes

The collective responses of neuroglial genes to ethanol and/or NNK exposures were more variably patterned than those observed for oligodendrocyte and transcription factor genes. The reductions in GFAP, NTRK, and GPD1 with ethanol and/or NNK exposures relative to control potentially carry significance with respect to impairments in blood-brain barrier integrity (GFAP), synaptic plasticity (NTRK)^[58]^, and oligodendrocyte responses to stress (GPD1). NNK’s stimulatory, and ethanol + NNK’s further up-regulation of CSPG4 suggests that NNK exposures during development inhibit neurite outgrowth and promote growth cone collapse during axon regeneration^[59,60]^ in adolescent brains, and that these effects are exacerbated by dual exposures to ethanol and NNK. These findings correspond with NNK’s inhibition of NCAM1expression, which mediates neuronal adhesion and neurite outgrowth^[58,61]^. It is also noteworthy that the ethanol-only effects were opposite those of NNK with respect to these genes, as well as GSTP1 (up-regulated by NNK and not ethanol) which negatively regulates p25-mediated neurodegeneration^[62]^, highlighting the divergent effects of these agent exposures on subsequent CNS molecular pathology. The overall findings with respect to NNK’s effects on CSPG4, NCAM1, and NTRK2 suggest that NNK exposures via tobacco smoke late in development has long-lasting deleterious effects on neuronal plasticity and repair in the adolescent brain.

### Transcription factors

The main findings with respect to transcription factors were that the expression levels of FOXO1, FOXO4, NKKX2-2 NKX6-1, Olig1, and Olig2 were increased by ethanol and/or NNK exposures, and for the most part, further sharply increased by ethanol + NNK exposures, reflecting additive or synergistic effects. Opposite effects occurred with respect to PAX6 and SOX9. Since both FOXO1 and FOXO4 target insulin signalling^[63–65]^, and NKX2-2 controls genes that have important roles in axonal guidance^[66]^, the findings suggest that the observed modulations in transcription factor gene expression may represent compensatory efforts to attenuate effects of impaired neuronal growth and plasticity, oxidative stress, and insulin resistance^[63,65]^. However, the inhibitory effects on PAX6 and SOX9 suggest that ethanol and NNK impair mechanisms of glial progenitor cell maintenance and eventual maturation^[67–70]^, and thereby contribute to white matter pathology in “FASD” that is mediated by developmental exposures to alcohol and/or tobacco smoke (NNK).

**Figure 6:**
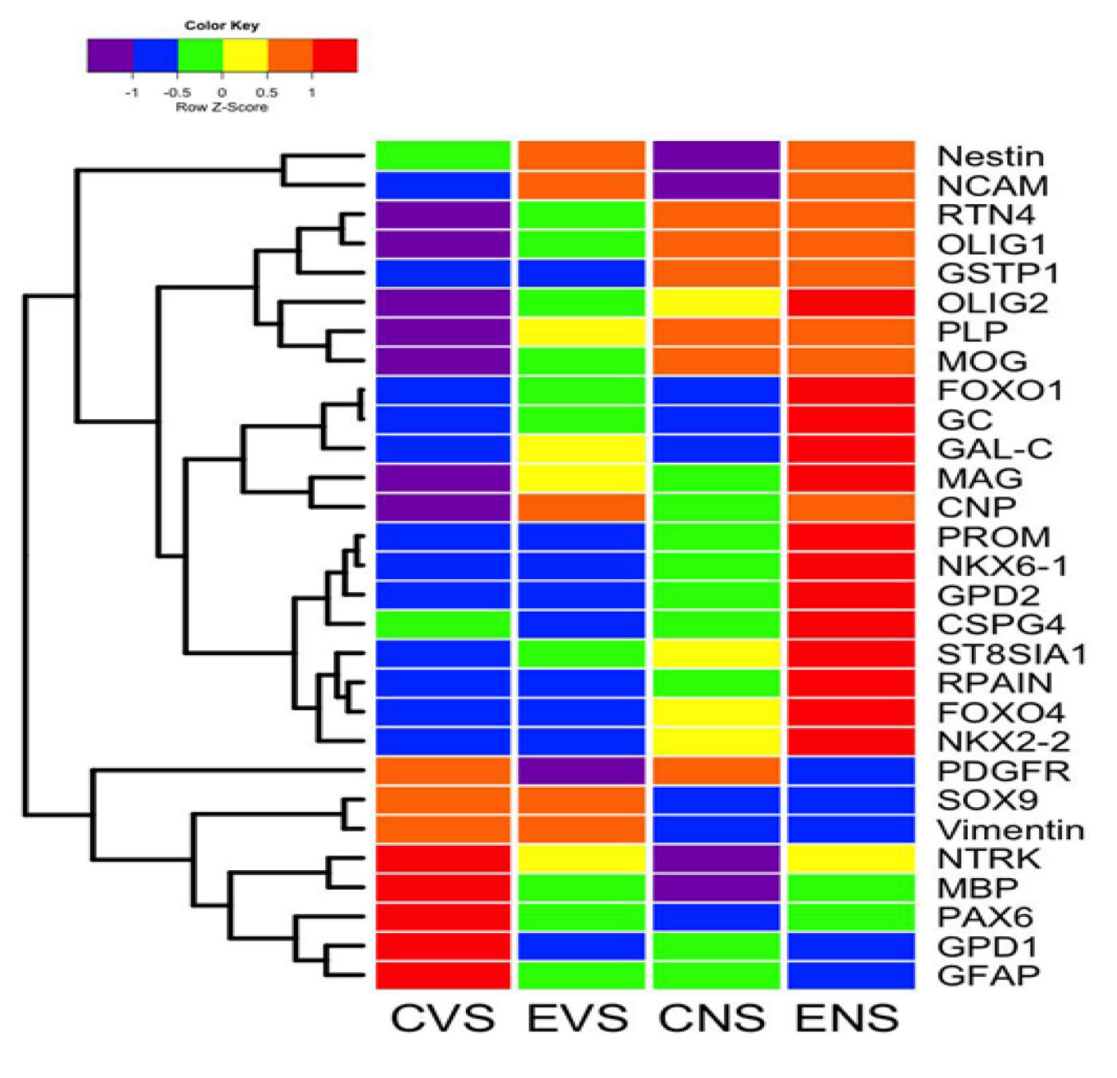
Heatmap illustrating hierarchical clustering of gene expression patterns across experimental groups. The heatmap was generated using Version 3.1 of R software. Results shown with the 6-color palette correspond to z-scores, which were scaled to have a mean of 0 and S.D. of 1. A hierarchical clustering algorithm was applied using the Euclidean distance function on the overall table to display a dendrogram of mRNAs encoding immature oligodendroglial genes, mature oligodendroglial genes, neuroglial genes, and transcription factors (See Figure 3). CVS = control diet with vehicle treatment and saline binge treatment; EVE = ethanol diet with vehicle treatment and ethanol binge; CNS = control diet with NNK treatment and saline binge treatment; ENE = ethanol diet with NNK treatment and ethanol binge.

Main effects of ethanol and NNK revealed by hierarchical clustered analysis of gene expression. Hierarchical clustering by heatmap analysis revealed broad independent and additive or synergistic effects of ethanol and NNK exposures on temporal lobe gene expression (Figure 6). Two dominant clusters were identified. The upper cluster (a) revealed predominantly low levels of gene expression in control brains and sharply elevated levels in ethanol + NNK exposed brains, whereas the lower dominant cluster (b) had opposite trends. Most of the genes in the lower cluster have important roles in maintaining structural integrity of white matter and synapse integrity in mature brains, whereas most of the genes in the upper cluster participate in various aspects of neuroglial, particularly oligodendrocyte structure and function during development. Concerning the up-regulation of immature neuroglial genes, with the exception of Nestin, NCAM, FOXO1, GC, and GalC, the dominant effects were largely driven by NNK and either maintained or further driven by combined ethanol + NNK exposures. Although the expression of PDGFR-α, SOX9, vimentin, NTRK, MBP, PAX6, GPD1, and GFAP were down-regulated by ethanol and/or NNK, the inhibitor effects were mainly or more prominently caused by NNK, with the exceptions of PDGFR-a and GPD1. Altogether, these analyses highlight the broader effects of late trimester NNK versus ethanol exposures on later development and maturation of white matter in the temporal lobes. In addition, these findings help delineate differential and additive or synergistic effects of developmental ethanol and NNK exposures on white matter structure and function in adolescent brains, and thus begin to explain how these factors contribute to brain pathology and sustained cognitive deficits in FASD.

## Supplementary Material
