# Supplementary material for: Potential Contributions of the Tobacco Nicotine-Derived Nitrosamine Ketone to White Matter Molecular Pathology in Fetal Alcohol Spectrum Disorder
Source: Int J Neurol Brain Disord. Author manuscript; Available in PMC 2017 Aug 30. (PMC5575815; doi:10.15436/2377-1348.16.729)
Supplement: Supplementary file 1 [file NIHMS896491-supplement-supplement_1.pdf]

Supplementary Table 1: PCR Targeted Array Results. Two-way ANOVA Summary of Ethanol and NNK effects on Oligodendroglial Gene Expression.

Protein	Ethanol Effect		NNK Effect		Ethanol x NNK Effect	
	F- Ratio	P- Ratio	F- Ratio	P- Ratio	F- Ratio	P- Ratio
**Immature Oligodendroglia**						
**Nestin**	4.800	0.059	1.200	N.S	1.200	N.S
**Vimentin**	0.290	N.S	80.18	< 0.0001	0.009	N.S
**PROM1**	576.3	< 0.0001	1065	< 0.0001	457.6	< 0.0001
**CNP**	0.350	N.S	4.710	0.062	0.198	N.S
**PDGFR-α**	0.637	N.S	12.71	0.007	0.139	N.S
**GC**	8.533	0.019	16.13	0.004	4.800	0.059
**GalC**	55.25	< 0.0001	198.7	< 0.0001	16.12	0.004
**Mature Oligodendroglia**						
**PLP**	4.670	0.063	15.63	0.004	5.512	0.047
**MOG**	0.614	N.S	7.445	0.026	1.752	N.S
**MAG-1**	7.588	0.025	3.247	0.109	0.324	N.S
**MBP**	0.066	N.S	5.792	0.043	5.687	0.044
**RTN4**	1.345	N.S	13.11	0.007	1.209	N.S
**RPAIN**	177.8	< 0.0001	455.1	< 0.0001	144.0	< 0.0001
**ST8sia1**	7.840	0.023	10.24	0.013	2.560	N.S

Temporal lobe RNA was analyzed using a targeted PCR array to examine ethanol, NNK and ethanol x NNK interactive effects on Oligodendroglia gene expression. Data were analyzed by Tow-way ANOVA with post-hoc Fisher's test. Data are graphed in Figure 1 - 3.

Back to Top: "↑"

Supplementary Table 2: PCR Targeted Array Results. Two-way ANOVA Summary of Ethanol and NNK effects on Neuroglial and Transcription Factor Gene Expression.

Protein	Ethanol Effect		NNK Effect		Ethanol x NNK Effect	
	F- Ratio	P- Ratio	F- Ratio	P- Ratio	F- Ratio	P- Ratio
**Neuroglial Markers**						
**CSPG4**	1.642	N.S	4.252	0.073	2.612	N.S
**GFAP**	8.237	0.021	12.18	0.008	3.518	0.098
**NCAM**	6.223	0.037	0.004	N.S	0.223	N.S
**NTRK2**	3.366	0.104	104.5	< 0.0001	111.4	< 0.0001
**GSTP1**	0.001	N.S	63.87	< 0.0001	0.178	N.S
**GPD1**	0.105	N.S	0.039	N.S	0.421	N.S
**GPD2**	44.38	0.0002	61.39	< 0.0001	38.59	0.0003
**Transcription Factors**						
**FOXO1**	29.79	0.0006	16.04	0.004	9.729	0.014
**FOXO4**	14.38	0.005	55.18	< 0.0001	10.83	0.011
**NKX2-2**	2.344	N.S	18.24	0.003	2.344	N.S
**NKX6-1**	425.0	< 0.0001	745.4	< 0.0001	277.0	< 0.0001
**Olig1**	0.432	N.S	8.106	0.022	0.299	N.S
**Olig2**	0.301	N.S	.590	N.S	0.004	N.S
**PAX6**	8.820	0.018	69.62	< 0.0001	30.42	0.0006
**SOX9**	0.021	N.S	17.66	0.003	0.173	N.S

Temporal lobe RNA was analyzed using a targeted PCR array to examine ethanol, NNK and ethanol x NNK interactive effects on Oligodendroglial Gene expression. Data were analyzed by Two-way ANOVA with post-hoc Fisher's test. Data are graphed in Figure 4 - 6.

Back to Top: "↑"
